# Effects of Soccer Cleat Stud Patterns Worn Versus Lower Extremity Injuries in the English Premier League

**DOI:** 10.1177/23259671251353762

**Published:** 2025-07-07

**Authors:** Sabrina M. Pescatore, Sterling J. DeShazo, William M. Weiss

**Affiliations:** † John Sealy School of Medicine, The University of Texas Medical Branch at Galveston, Galveston, Texas, USA; ‡ Assistant Professor, Department of Orthopaedic Surgery and Rehabilitation, The University of Texas Medical Branch at Galveston, Galveston, Texas, USA; Investigation performed at The University of Texas Medical Branch at Galveston, Galveston, Texas, USA

**Keywords:** athletic injuries, European football, footwear, lower extremity, risk analysis, risk assessment, soccer

## Abstract

**Background:**

Soccer has driven increased player physical demands, resulting in elevated levels of lower extremity injuries, especially among elite players.

**Purpose:**

To analyze the potential effects of soccer cleat stud patterns worn by players who sustained lower extremity injuries in the English Premier League (EPL).

**Study Design:**

Cohort study; Level of evidence, 3.

**Methods:**

Cleat models, 510 players, and 221 injuries were analyzed from the EPL 2021-2022 season. The injured player cohort was established using Transfermarkt.com, and cleat stud patterns—categorized as very aggressive (VA), mildly aggressive (MA), and nonaggressive (NA)— were obtained from footballbootsdb.com, product websites, and online reviews. Odds ratios (ORs) and 95% CIs were used to assess the risk of lower extremity injury. Poisson regression, multinomial linear regression, chi-square tests, and *t* tests were used to assess the relationships between age, position, cleat type, and injuries.

**Results:**

VA stud patterns had significantly higher injury odds compared with other models (OR_VA_, 1.362 [95% CI, 1.008-1.842]; *P* = .023), while MA stud patterns had significantly lower injury odds (OR_MA_, 0.579 [95% CI, 0.352-0.952]; *P* = .016). NA stud patterns were not significantly associated with the odds of injury. Cleat type and player position together were significantly associated with differences in observed versus expected injuries (χ^2^ = 37.89; *P* < .0001; *df* = 6). Players aged between 23 and 30 years had a significantly higher incidence of injury compared with players aged 18 to 22 years (β_23-26-year-olds_ = 0.4205; *P* = .03; β_27-30-year-olds_ = 0.5199; *P* = .006).

**Conclusions:**

EPL players wearing VA stud patterns may have a higher risk of lower extremity injuries compared with those with MA or NA patterns. Older age and player position, combined with cleat type, were associated with increased risk of injury.

As the most popular sport in the world, soccer has become faster, more powerful, and more intense over time, requiring players to constantly elevate both their general athletic and technical skills.^19,21^ The growing intensity of the game is thought to be directly correlated with the increasing incidence of player injuries, especially at elite levels, where the unrelenting physical demands during the season make the players very susceptible to injuries.^10,19^ It is vitally important for soccer clubs to mitigate player injury risk as much as possible. The loss of key players can not only negatively affect the overall success of a team but can also pose financial burdens; for instance, each English Premier League (EPL) team loses a mean of £45 million per year because of injuries.^11^

Some research suggests that in men’s professional soccer, lower extremity injuries have the highest incidence rates of any injury type.^26^ One area of potential contribution to these lower extremity injuries is the soccer shoe or cleat, which is the primary interface between the player and the playing surface. Numerous cleat-specific biomechanical studies have assessed the effect of cleat characteristics on the game of soccer and their contribution to injuries, including playing surface interactions.^3,8,21,24^ However, although epidemiological research has analyzed soccer injury prevalence, no connection has been established between injury prevalence and the cleat stud patterns worn by players at the time of injury.

Previous studies have correlated soccer injuries to player age, exercise load, level or intensity of play, previous injuries, and the standard of training.^32^ No current literature has attempted to assess cleat stud patterns worn versus lower extremity injury prevalence in the EPL. This study aimed to investigate the potential effects of cleats with differing stud patterns worn versus lower extremity injuries sustained by players, using injury prevalence and injury category data from the EPL. We hypothesized that certain cleat stud patterns would be associated with overall lower extremity injuries and specific injuries experienced by EPL players.

## Methods

### Player, Injury, and Cleat Attribute Data Collection

Player data, including player age and position, was collected retrospectively from the 20 teams in the EPL 2021-2022 season, which spanned from August 13, 2021, to May 22, 2022. Out of a total of 804 players, we excluded 294 players who did not meet the minimum of 1 game appearance and ≥15 minutes of playing time, as determined by EPL statistics and Transfermarkt.com, a large, European-based website specializing in soccer-related news, scores, and various statistics, including player injuries.^33,42^ Collection of data across an entire season with a focus on active players enabled our analysis to be representative of overall EPL league conditions and player performance.

For the 510 active players, we identified 221 players with lower extremity injuries, after excluding 4 players whose cleat models at the time of injury could not be independently verified. Each injury occurrence was counted as a separate datapoint, including multiple injuries from a single player. The 221 player injuries were then separated into 7 categories: ankle, calf, foot, hamstring, knee, lower leg, and thigh. Players with upper extremity injuries, injuries of the pelvis, groin, or hip, or nonspecified conditions were excluded.

The cleats worn by injured players were determined using footballbootsdb.com, a publicly available database that tracks soccer statistics from the top European-based leagues and major tournaments such as the World Cup. Cleat data were not collected for noninjured players. If a player wore multiple cleat models throughout the season, final confirmation was achieved using the player’s or the club’s social media accounts and the date of injury.^14^ If social media posts did not have the injury date, separate internet searches were executed to find images from the specific game where the injury occurred. If a single player had multiple injuries, their cleats were checked and confirmed through their social media accounts and the footballbootdb.com database, with the corresponding injury date. For the 221 players with lower extremity injuries, 15 cleat models were analyzed.

**Figure 1.**
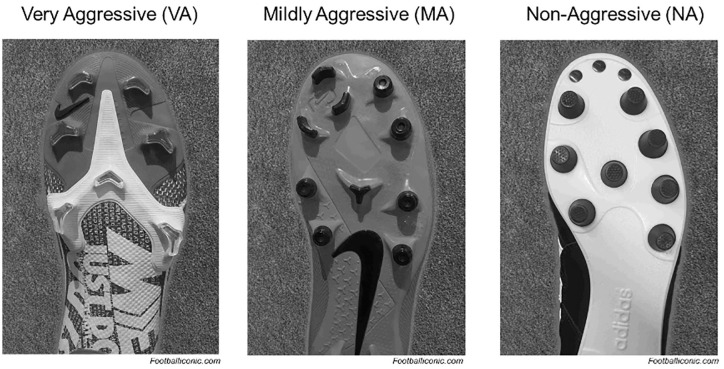
Visual demonstration of cleat stud pattern. Image courtesy of footballliconic.com.

Once identified, the stud pattern and geometry for each cleat model worn by the 221 injured players were collected and confirmed using product websites and online third-party cleat reviews.^7,15,38,39^ Using this data, the cleats were classified into 3 widely accepted categories previously established in other research studies and used descriptively in cleat reviews.^16,20,36^ Cleats with chevron or bladed studs on both the forefoot and hindfoot of the shoe were defined as “very aggressive (VA).” Cleats with chevron or bladed studs on either the forefoot or the hindfoot, but not both, were defined as “mildly aggressive (MA).” Cleats using only conical or rounded studs on the forefoot and hindfoot with no chevron or bladed studs were defined as “nonaggressive (NA)” (Figure 1).^28^

### Statistical Analyses

Odds ratios (ORs) and confidence intervals were utilized to determine the odds for potential lower extremity injury while wearing a particular cleat model and stud pattern.^1,40^ The cleat stud pattern was set as the experimental condition (cleat stud pattern worn or not worn), with injury as the outcome (injury or no injury). In total, 20 ORs were calculated: 3 comparing stud patterns worn to total injuries and 17 comparing stud patterns worn to specific injuries.

All OR calculations were assessed for their confidence intervals and their significance, which was performed by a 1-tailed *z*-score assessment because of the small sample size. Significance was set at *P* < .05. All 15 cleat models were included when analyzing stud patterns for both total injuries and specific injuries. ORs for stud patterns were directly compared with each other by converting ORs to log ORs.^5,34^

Age and player position were analyzed for overall injuries, specific injuries, and cleat types worn using Poisson regression, multinomial logistic regression, chi-square tests of independence with standardized residuals analysis, and pairwise *t* tests via Python programming software Version 3.10.

## Results

### EPL Stud Pattern Worn and Total Lower Extremity Injuries

For the EPL 2021-2022 season, the stud pattern and injury data for the 510 active players included in our analysis are summarized in Table 1. The most popular stud pattern worn was VA, followed by NA and MA. When correlated with the 221 player injuries, the stud pattern with the highest injury prevalence was VA, followed by NA and MA. The total league-wide injury prevalence was 43.33%.

**Table 1:** Stud Pattern Worn Versus Total Lower Extremity Injuries During the EPL 2021-2022 Season^*a*^

EPL Stud Pattern 2021-22	Prevalence in EPL Players	Prevalence of Lower Extremity Injuries in EPL Players
VA	304	141
MA	83	27
NA	123	53
Total	510	221

a EPL, English Premier League; MA, mildly aggressive; NA, nonaggressive; VA, very aggressive.

**Figure 2.**
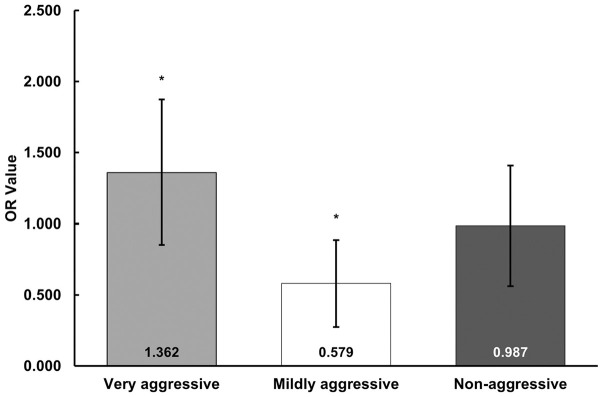
Odds ratios, standard errors, and injury significance for stud pattern worn versus total lower extremity injuries.

ORs for VA stud patterns were significantly associated with higher odds of injury (OR_VA_, 1.362; [95% CI, 1.008-1.842]; *P* = .023), MA stud patterns were significantly associated with lower odds of injury (OR_MA_, 0.579 [95% CI, 0.352-0.952]; *P* = .016), and NA stud patterns were not significantly associated with higher or lower odds of injury (OR_NA_, 0.969 [95% CI, 0.655-1.487]; *P* = .238) (Figure 2).

To compare calculated ORs between different stud pattern types, log odds were used for direct comparison. VA stud patterns were found to be significantly different from MA stud patterns (*z*-statistic, 2.73; *P* = .0063). All other log OR direct comparisons were not significant.

### EPL Stud Pattern Worn and Specific Lower Extremity Injuries

The 3 stud patterns worn and 221 lower extremity injuries were further categorized into specific lower extremity injuries (Table 2). The most prevalent specific lower extremity injury was the hamstring, followed by the knee, ankle, thigh, calf, foot, and lower leg.

**Table 2:** Stud Pattern Worn Versus Total Lower Extremity Injuries During the EPL 2021-2022 Season^*a*^

EPL Stud Pattern 2021-22	Prevalence of Specific Lower Extremity Injuries in EPL Players							
	Ankle	Calf	Foot	Hamstring	Knee	Lower Leg	Thigh	Total
VA	30	13	15	32	36	1	14	141
MA	6	6	0	8	6	0	1	27
NA	9	5	1	16	10	2	10	53
Total	45	24	16	56	52	3	25	221

a EPL, English Premier League; MA, mildly aggressive; NA, nonaggressive; VA, very aggressive.

When ORs were calculated for each stud pattern worn versus specific lower extremity injuries in EPL players, none of the ORs were significant. However, the highest observed ORs by cleat stud type included players sustaining an ankle or knee injury with VA stud patterns (OR_ankle_, 1.394; OR_knee_, 1.595), and players sustaining a hamstring injury with NA stud patterns (OR_hamstring_, 1.297).

### EPL Player Age and Position and Versus Total and Specific Lower Extremity Injuries

To assess the potential relationships between player age and total injuries, a Poisson regression was used. Players were grouped by age into 5 categories and analyzed: 18-22 years (n = 47), 23-26 years (n = 62), 27-30 years (n = 73), 31-35 years (n = 39), and 36-40 years (n = 4). Using the 18-22 years age group as a baseline reference group, the 23-26 years age group had a significant and positive coefficient (β coefficient, 0.4205; *P* = .03), indicating a 52.2% increase in injuries compared with the reference group. The 27-30 years age group also had a significant and positive coefficient (β coefficient, 0.5199; *P* = .006), indicating a 68.2% increase in injuries compared with the reference group. The 31-35 years age group had a negative but nonsignificant coefficient compared with the reference group (β coefficient, −0.0230), indicating a slight decrease of 2.3% in injuries compared with the reference group. The 36-40 years group could not be accurately assessed because of the small sample size.

To assess the potential relationships between player age and specific injury type, a multinomial logistic regression was used. The analysis included a total of 221 players with a log likelihood ratio of −382.17 versus the null model ratio of −386.41 (*P* = .205), suggesting that overall, player age is not a significant predictor of injury type as observed in this dataset.

Age was significantly associated with calf injuries (*P* = .008), where each additional year of age in players increased their log odds risk of sustaining a calf injury by 0.1687. Although not statistically significant (*P* = .057), hamstring injuries had a positive association with age, where each additional year of age in players increased their log odds risk of sustaining a hamstring injury by 0.0951. All other specific injuries, including foot, knee, lower leg, and thigh, were not significantly associated with age.

The relationship between cleat stud type and mean age among injured players was analyzed via pair-wise *t* tests. VA cleat stud patterns had a mean age of 26.35 years, NA cleat stud patterns had a mean age of 28.34 years, and MA cleat stud patterns had a mean age of 26.63 years. A significant difference was found between the mean ages of VA and NA cleat stud patterns (*P* = .0129). No significant differences were found between the mean ages of injured players wearing MA versus NA or MA versus VA cleat stud types.

The potential relationship between player position (defender, midfielder, forward, and goalkeeper as reference category) and specific lower extremity injuries was also assessed via multinomial logistic regression. The analysis included a total of 221 players with a log likelihood ratio of −378.03 compared with the null model of −386.41 (*P* = .540), also suggesting that player position is not a significant predictor of injury type. No significant associations were found between player position (defender, midfielder, and forward) compared with specific injuries of ankle, calf, foot, hamstring, knee, lower leg, or thigh (*P* > .05 for all groups).

### EPL Position and Stud Patterns and Total Lower Extremity Injuries

A chi-square test of independence was used to assess the effect of player position + cleat type worn versus injury rates sustained. Because positional cleat data for the remaining 285 uninjured players was not available, the prevalence of each cleat stud type per position within the EPL league was calculated for defenders, midfielders, forwards, and goalkeepers. Observed injury counts were categorized by position and cleat type (Table 3). Using the chi-square analysis, expected injury counts were calculated for each position and cleat type and compared with observed outcomes for deviations. Expected injury counts represent how many injuries would be expected by player position and cleat type if no association existed between these variables. To further delineate the observed and expected count interactions by cell, standardized residuals between observed and expected injuries were calculated for each cleat type and position.

**Table 3:** Observed and Expected Injuries Based on Cleat Type and Position in EPL Players During the 2021-2022 Season^*a*^

Cleat Type	Position	Observed Injuries	Expected Injuries	Standardized Residuals
VA	Forward	49	35.09	2.35^*b*^
	Midfielder	47	44.66	0.35
	Defender	42	56.14	–1.89
	Goalkeeper	3	5.10	–0.93
MA	Forward	0	6.72	–2.59^*b*^
	Midfielder	6	8.55	–0.87
	Defender	21	10.75	3.13^*b*^
	Goalkeeper	0	0.98	–0.99
NA	Forward	6	13.19	–1.98^*b*^
	Midfielder	17	16.79	0.05
	Defender	25	21.10	0.85
	Goalkeeper	5	1.92	2.22^*b*^

a EPL, English Premier League; MA, mildly aggressive; NA, nonaggressive; VA, very aggressive.

b Standardized residual calculations are considered significant if the value exceeds ±1.96.

The chi-square statistic indicated significant deviation between expected and observed counts within the dataset (χ^2^ = 37.89; *P* < .0001; *df* = 6). With VA cleats having significantly higher than expected injury rates in forwards, NA cleats having significantly higher than expected injury rates in goalkeepers and significantly lower than expected injury rates in forwards, and MA cleats having significantly higher than expected injury rates in defenders but significantly lower than expected injury rates in forwards (see Table 3).

## Discussion

This study provides unique insight into the potential relationship between cleat stud patterns and lower extremity injuries. Of the 3 stud patterns worn by injured EPL players, VA demonstrated significantly higher ORs correlated with total lower extremity injuries, while MA demonstrated significantly lower ORs. Research has shown that VA stud patterns designed to improve grip and produce higher friction for playing on grass surfaces tend to lower the overall stability of the cleat due to increased lateral loading.^3,29^ The proposed mechanisms by which this occurs include resistance to rotation and increasing rotational torque.^4,37^

Biomechanical studies have determined that VA stud patterns increase traction and rotational torque, which raises the risk of lower limb injury.^16,26,44^ Although some traction is necessary to optimize performance, excessive traction elevates the risk of lower extremity injury.^35,36^ Our results, which showed that VA stud patterns were more correlated with injury than MA or NA stud patterns, support these previous findings. Conversely, insufficient traction has also been implicated in injury risk, leading to suboptimal playing and potential for slippage.^31^ Although we found that NA stud patterns were not significantly different from VA or MA, they were more strongly correlated with injuries than MA stud patterns. Additional epidemiological studies are needed to investigate whether wearing MA stud patterns in competitive play could potentially mitigate total lower extremity injuries.

Of the 3 stud patterns worn by injured EPL players, none had significant correlations with specific lower extremity injuries. The odds of a player sustaining a hamstring injury were higher in NA stud patterns (OR_hamstring_, 1.297), while the odds of a player sustaining an ankle or knee injury were highly associated with VA stud patterns (OR_ankle_,1.394; OR_knee_, 1.595). We believe an analysis with a larger cohort is necessary to improve the power and precision of these estimates. A larger dataset analysis would allow for greater insights as to whether specific injuries are correlated with particular stud patterns and should be conducted in future studies, given the observed trends.

Player position has been previously defined as a relevant risk factor for injuries.^2,17^ The current literature disagrees on positional associations with injury rates in the elite leagues, with different injury rates being reported by position.^2,6,12,25^ The Italian League has reported that all field position players are equally affected by injuries, whereas the French League and German League report that forwards and midfielders have the highest injury rates, respectively.^2,6,12,25^ Player position and injury rates are thought to be associated with the variable movements and physiological demands required by respective positions.^2,6^ For example, defenders spend significantly less time sprinting and running compared with midfielders and forwards but spend more time jumping and moving backwards, moving laterally, and engaging in feet-first tackles.^6^ Midfielders spend the most time running and sprinting, cover the greatest distances, and have more forward and diagonal or arc-shaped runs.^6^ Forwards engage in the most physical contact, sprint in the forward direction, and exhibit rapid and high-intensity changes in direction.^6^ Therefore, players may select cleat types that they believe will best suit their needs anatomically and positionally.^18,36^ Our results are consistent with previous literature,^2,25^ which indicates defenders sustain the highest percentage of injuries in the EPL, with 39.82% of observed injuries in the 2021-2022 season being composed of defenders, followed by midfielders (31.67%), forwards (24.89%), and goalkeepers (3.62%). Although defenders had the highest injury rates overall, no significant differences were found between player position and specific injury type. However, significant differences were found between observed and expected overall injuries when analyzing cleat stud type worn in combination with player position.

Cleat model selections in elite leagues, such as the EPL, are likely influenced by several factors, including individual and club-based sponsorship agreements, club-specific policies, and individual player preferences.^6,18,36^ Many players who have sponsorship contracts are required to wear cleats provided by their respective sponsors, which can limit their options depending on the brand and model their sponsors provide. Clubs may also influence cleat selection, particularly if they have their sponsorship agreements or have guidelines for cleat types based on match playing surfaces or training conditions.^36^ In addition, players themselves may select cleats based on their perceptions of the performance advantage a cleat model may provide. For example, VA stud patterns are designed to enhance traction, agility, and explosiveness in changes of direction, which may be appealing to forwards, whose position typically has more high-intensity, fast-paced position changes than defenders.^6^ Players may also select cleats based on anatomical comfort. A player with wider feet will likely not choose to wear historically narrow models, which may inevitably limit their selections to models that have specific stud types. These factors and perceptions could explain why VA cleats were the most worn across all positions, despite their possible link to certain injury types. Future studies could explore the role of individual and club-wide sponsorships, club policies, and player preferences in cleat selection to better understand how cleat choices affect both performance and injury risk.

Overall, the chi-square statistic identified that positional injuries were significantly associated with a player’s cleat type. VA cleats were associated with significantly higher than expected injury rates in forwards, slightly higher than expected injury rates in midfielders, slightly lower than expected rates in goalkeepers, and much lower than expected rates in defenders. NA cleats were associated with significantly higher than expected injury rates in goalkeepers, slightly higher than expected injury rates in defenders, no difference in midfielders, and significantly lower than expected rates in forwards. MA cleats were associated with significantly higher than expected injury rates in defenders only, slightly lower than expected rates in midfielders and goalkeepers, and significantly lower than expected rates in forwards (see Table 3). Taken together, these comparisons highlight the relationship between cleat stud type and injury rates across different positions. Specifically, VA cleats, although highest in prevalence for forwards, midfielders, and defenders, were only associated with significantly higher observed injury rates in forwards. NA and MA cleats, although lower in prevalence than VA cleats for forwards, midfielders, and defenders, demonstrated significantly lower observed injury rates for forwards only but significantly increased risks in goalkeepers (NA) or defenders (MA). These results highlight the potential influence of player position and cleat stud types on injuries. While this study identifies significant trends, it is important to note that its goal was to identify general patterns, rather than overinterpret relationships that could potentially be significant due to sample-dependent findings. To further explore these results and draw more definitive conclusions on the associations between position injuries and specific cleat types, future analyses should collect data across multiple seasons and leagues to evaluate possible existing relationships for increased risks or protective effects of cleat type versus positional injuries.

In addition to player position, increasing age has also been identified as a risk factor for injuries.^2,17^ Physiological condition of athletes changes with increasing age, including changes in muscle fiber composition, muscle strength, healing ability, and decreases in range of motion and flexibility, therefore potentially increasing players’ susceptibility to certain injuries.^9,13,23,27^ The mean age of injured players wearing NA cleat stud patterns was significantly higher than the mean age of injured players wearing VA cleat stud patterns (28.34 vs 26.35; *P* = .0129). Further, we found that players in the 23-26 years age group and 27-30 years age group were 52.2% and 68.2% significantly more likely to sustain an injury overall when compared with the 18-22 years age group, respectively. Players in the 31-35 years age group were 2.3% less likely to sustain an injury compared with the 18-22 years age group, although this was not significant and likely due to fewer players in this age range playing at the elite level. When analyzing specific injuries, increasing age was significantly associated with calf injuries (*P* = .008) and strongly correlated with hamstring injuries (*P* = .057). Meaning, with each additional year of age, a player increases their risk of sustaining a calf injury and hamstring injury by 18.4% and 10%, respectively.

Recent literature indicates that hamstring injuries have doubled from 12% to 24% over the last 21 years in the top European league, the United European Football Association.^10^ This is consistent with our data, where hamstring injuries accounted for 25.34% of all EPL player lower extremity injuries in the 2021-2022 season. This may be caused by the increasing intensity of soccer and the number of matches played overall.^10^ Although not statistically significant (*P* = .057), our data also support previous studies that link hamstring injuries with increasing player age.^17^ The higher incidence of hamstring injuries warrants further evaluation of hamstring injury causes, especially when considering the potential injury mitigation of MA cleats.

Previous studies suggest that lighter, more mobile cleats may sacrifice ankle support for lower weight, resulting in a higher incidence of ankle injuries.^30^ Our results showed that VA stud patterns, which normally utilize lighter, more mobile material, had a stronger association with ankle injury prevalence. We hypothesized that both increased lateral plantar loading along less ankle stability would lead to a higher ankle injury incidence in VA stud patterns. To mitigate ankle-specific injuries, the current literature suggests that using external ankle support, such as taping or a brace, can help prevent musculoskeletal injuries.^43^

Research shows that higher torsional and rotational forces increase joint load to the knee, placing a player at a higher risk for anterior cruciate ligament injury.^22^ Our results indicated no significant association of knee injury risk with the use of rounded studs in MA or NA stud patterns; however, there was a strong but nonsignificant association between knee injuries and VA stud patterns. This higher incidence of knee injuries with VA stud patterns could be related to the increased rotational traction, something previously correlated with knee-specific injuries in American football.^41^

Although not statistically significant, we believe that these strong associations with ankle, hamstring, and knee-specific injuries further highlight the potential injury risk of VA and NA stud patterns. Further biomechanical research is suggested to address these possible associations via multileague or multiseason studies that allow for larger cohorts and therefore more powerful and representative statistical analyses.

### Limitations

We acknowledge that there may be inherent errors in our source data. First, Transfermarkt.com is not a medical database nor is it officially team sponsored, often grouping injuries into broad categories such as ankle, calf, et cetera, versus indicating the specific anatomy injured for players. This limited our ability to effectively correlate specific injury types with stud patterns. We attempted to mitigate the possibility of injury data error by confirming player injuries reported in Transfermarkt.com with player social media posts and internet searches. Next, we utilized the footballbootsdb.com website for soccer cleat attribute data, as no peer-reviewed, standardized database was available. We attempted to mitigate potential errors by confirming attribute data with product websites and online reviews.^14,42^ However, this may have impacted the stud pattern classifications of the 15 cleat models. We could not determine a definite cleat stud type for uninjured players’ positions; therefore, proportions were utilized for the remaining 285 uninjured players in the EPL who did not sustain an injury for the purpose of statistical analysis. By excluding injuries other than lower limb injuries, we did not consider any additional risks of upper limb, spinal, head, or neck injuries to players as a consequence of losing their footing due to poor foot anchorage. We did not factor in the potential risks of artificial turf risk to EPL players who may play on hybrid grass/artificial pitches. Finally, our focus on elite-level soccer players and their injuries may not apply to soccer athletes playing at lower levels. Future studies should consider the effect of cleat stud patterns/additional cleat attributes alone and in combination with other factors such as position and age versus lower extremity injuries sustained by players using medical injury databases, multileague data collection, and/or sanctioned injury data obtained directly from the teams.

## Conclusion

While preliminary, our analysis suggests that highly significant associations may exist between some cleat stud patterns and models worn by EPL players in the 2021-2022 season versus total and specific lower extremity injuries. We found that VA stud patterns were significantly associated with increased odds of lower extremity injury, while MA stud patterns were significantly associated with decreased odds of lower extremity injury. VA stud patterns were also significantly more associated with lower extremity injuries when directly compared with MA stud patterns via log odds ratios. Increasing player age was associated with increased overall and some specific injuries. When combined with the cleat stud type, position was significantly associated with overall injuries. EPL clubs and players should consider whether a particular stud pattern is potentially contraindicated for injury risk alone and in combination with player age and position.
